# A functional magnetic resonance imaging study of frontal networks in obsessive-compulsive disorder during cognitive reappraisal

**DOI:** 10.1192/j.eurpsy.2022.2322

**Published:** 2022-10-03

**Authors:** Víctor De la Peña-Arteaga, Pedro Morgado, Beatriz Couto, Sónia Ferreira, Inês Castro, Nuno Sousa, Carles Soriano-Mas, Maria Picó-Pérez

**Affiliations:** 1 Psychiatry and Mental Health Group, Neuroscience Program, Institut d’Investigació Biomèdica de Bellvitge – IDIBELL, L’Hospitalet de Llobregat, Barcelona, Spain; 2 Department of Clinical Sciences, School of Medicine, Universitat de Barcelona – UB, L’Hospitalet de Llobregat, Barcelona, Spain; 3 Life and Health Sciences Research Institute (ICVS), School of Medicine, Universidade do Minho, Braga, Portugal; 4 ICVS/3B’s, PT Government Associate Laboratory, Braga/Guimarães, Portugal; 5 2CA-Clinical Academic Center, Braga, Portugal; 6 Network Center for Biomedical Research on Mental Health (CIBERSAM), Instituto de Salud Carlos III (ISCIII), Madrid, Spain; 7 Department of Social Psychology and Quantitative Psychology, Universitat de Barcelona – UB, Barcelona, Spain; 8 Departamento de Psicología Básica, Clínica y Psicobiología, Universitat Jaume I, Castelló de la Plana, Spain

**Keywords:** Cognitive reappraisal, emotion regulation, fMRI, frontal networks, OCD

## Abstract

**Background:**

Patients with obsessive-compulsive disorder (OCD) present difficulties in the cognitive regulation of emotions, possibly because of inefficient recruitment of distributed patterns of frontal cortex regions. The aim of the present study is to characterize the brain networks, and their dysfunctions, related to emotion regulation alterations observed during cognitive reappraisal in OCD.

**Methods:**

Adult patients with OCD (*n* = 31) and healthy controls (HC; *n* = 30) were compared during performance of a functional magnetic resonance imaging cognitive reappraisal protocol. We used a free independent component analysis approach to analyze network-level alterations during emotional experience and regulation. Correlations with behavioral scores were also explored.

**Results:**

Analyses were focused on six networks encompassing the frontal cortex. OCD patients showed decreased activation of the frontotemporal network in comparison with HC (*F*(1,58) = 7.81, *p* = 0.007) during cognitive reappraisal. A similar trend was observed in the left frontoparietal network.

**Conclusions:**

The present study demonstrates that patients with OCD show decreased activation of specific networks implicating the frontal cortex during cognitive reappraisal. These outcomes should help to better characterize the psychological processes modulating fear, anxiety, and other core symptoms of patients with OCD, as well as the associated neurobiological alterations, from a system-level perspective.

## Introduction

The neurobiological underpinnings of human emotion have been long studied from a neuroscience and neuroimaging perspective [1, 2]. More specifically, in recent years, considerable research efforts have been directed to explore the regulatory effects of frontoparietal cognitive control networks on subcortical emotional processing regions, describing alterations across major neuropsychiatric disorders [3, 4]. However, such alterations have been comparatively less studied in obsessive-compulsive disorder (OCD).

OCD patients are characterized for presenting difficulties in cognitive and emotional regulation [5–7]. Previous studies suggest that these patients might have difficulties activating frontoparietal networks when cognitive control is required [8], showing less recruitment of the dorsolateral prefrontal cortex (dlPFC) as well as diminished frontal-limbic connectivity [9, 10]. Although the pattern of alterations observed in patients with OCD may differ from what is observed in anxiety disorders in specific conditions, such as the implicit regulation of emotions during active responding, where limbic-prefrontal connectivity may be increased [11], such differences are more difficult to appreciate, for instance, during the anticipation of disorder unspecific emotional stimuli [12]. Alterations in the cognitive regulation of emotions in patients with OCD have been recently summarized in Ferreira et al. [13].

Network-based analyses are an interesting alternative to explore neurofunctional abnormalities in emotion regulation circuits in patients with OCD. These analyses provide a comprehensive description of alterations involving the coordinated action of different brain regions, beyond the mere description of regional-specific activation dysfunctions, and have been recently applied to characterize disruptions in emotion regulation circuits in addictive disorders [14]. More specifically, in view of the above reviewed literature, the examination of frontoparietal, frontotemporal, and frontolimbic networks seems to be of special interest to characterize alterations of emotion regulation networks in OCD. In this sense, one possible methodological approach able to capture alterations in cognitive control networks is independent component analysis (ICA), which has been used in the past to identify and evaluate networks mainly involving frontal regions [15]. ICA is a data-driven approach that assumes that source signals of functional magnetic resonance imaging (fMRI) data represent coherent groupings of magnetic resonance imaging (MRI) activations, which implies the representation of a functionally connected network. In task-fMRI studies, ICA allows to identify intrinsic functional connectivity networks and how the time courses associated with these networks are modulated by the task. This provides new insights into functional activity hidden from conventional voxel-wise general linear model (GLM) analyses [16, 17].

The aim of the present study is therefore to characterize the brain networks, and their dysfunctions, related to the (altered) emotion regulation phenotype observed in patients with OCD. For this, we explored OCD patients and healthy controls (HCs) with fMRI while performing a cognitive reappraisal protocol. Brain activity during this task was characterized at the network level by using an ICA approach, and we explored for potential between-group differences in the different functional networks (e.g., components) describing coordinated patterns of brain activity between frontal cortex regions and other brain areas. We hypothesized that patients with OCD will exhibit alterations in networks involving frontotemporal, frontoparietal, and frontolimbic regions during the cognitive regulation of emotion. We believe these outcomes can contribute to a better understanding of emotional processing difficulties in OCD.

## Methods

### Sample

A total of 67 adult (≥18 years) individuals (35 OCD patients and 32 HCs) participated in the study. Six participants, however, were excluded due to MRI artifacts or suboptimal task performance. The final sample consisted therefore of 31 patients with OCD (17 females; mean age = 30.00, SD = 11.12 years) and 30 HCs (16 females; mean age = 29.00, SD = 12.07 years). Patients were recruited at the Department of Psychiatry of *Hospital de Braga* (Braga, Portugal) and were diagnosed following DSM-5 criteria by an experienced psychiatrist. Additionally, the Mini-International Neuropsychiatric Interview [18] was administered to explore other potential psychopathological alterations. Exclusion criteria for patients included current presence of other psychiatric diagnoses (Axis I or Axis II disorders) or current or past presence of major neurological or medical conditions. Most patients (80.64%) were medicated at the time of recruitment, although treatments were kept constant throughout the study. Controls were recruited from the same sociodemographic setting and were excluded if they reported current or past presence of any psychiatric, neurological, or major medical condition, or if they reported current or past treatment with psychotropic medication. Participants from both groups were also excluded if they were not able to undergo the MRI exam, or if anatomical abnormalities were detected in the MRI scan. Table 1 summarizes clinical and sociodemographic information of study groups.

**Table 1.** Demographic and clinical characteristics of the sample.

	OCD (*N = 31*)	HC (*N = 30*)	Statistic (*p*-value)
Age, mean (SD)	30 (11.12)	29 (12.07)	*U* = 410.5 (0.435)
Sex/gender, *N* females (%)	17 (54.83)	16 (53.33)	χ^2^(1) = 0.01 (0.906)
Years of education, mean (SD)	13.03 (3.81)	13.80 (3.83)	*U* = 525 (0.385)
Age of onset, mean (SD)	17.38 (7.74)	—	—
Medication, *N* (%)			
SSRI	17 (54.83)	—	—
Tricyclic	2 (6.45)	—	—
SSRI + Tricyclic	5 (16.12)	—	—
SSRI + AP	1 (3.22)	—	—
Unmedicated	2 (6.45)	—	—
Naïve	4 (12.90)	—	—
Y-BOCS compulsions	13.74 (2.30)	—	—
Y-BOCS obsessions	11.96 (3.11)	—	—
Y-BOCS total	25.71 (4.93)	—	—
OCI washing	4.23 (3.45)	1.63 (1.99)	*U* = 237.5 (0.001*)
OCI checking	6.03 (3.78)	2.23 (2.09)	*U* = 178.5 (<0.001*)
OCI ordering	5.93 (3.70)	3.76 (2.48)	*t*(58) = −2.66 (0.010*)
OCI hoarding	3.46 (3.24)	3.33 (2.82)	*U* = 449.5 (1.0)
OCI obsessing	7.26 (3.68)	2.43 (2.62)	*U* = 131.5 (<0.001*)
OCI neutralizing	4.23 (3.80)	1.90 (1.93)	*U* = 309.5 (0.035*)
OCI total	30.93 (15.76)	15.43 (10.24)	*t*(58) = −4.51 (<0.001*)
ERQ reappraisal	26.19 (8.15)	29.36 (7.73)	*t*(59) = 1.55 (0.124)
ERQ suppression	14.74 (5.19)	14.70 (5.77)	*t*(59) = −0.03 (0.976)
Reactivity	1.92 (1.64)	2.52 (0.92)	*U* = 546 (0.157)
Success	0.31 (0.95)	0.87 (0.87)	*t*(57) = 2.34 (0.023*)

Note: Total N = 60 for the OCI subscales, N = 60 for the ratings’ reactivity variable, and N = 59 for the success variable. *Denotes statistical significance (P<0.05).

Abbreviations: AP, antipsychotics; ERQ, emotion regulation questionnaire; HC, healthy controls; OCD, obsessive-compulsive disorder; OCI, obsessive-compulsive inventory; SSRI, selective serotonin reuptake inhibitors; Y-BOCS, Yale-Brown obsessive-compulsive scale.

All participants provided written informed consent before starting the study procedures, which was conducted according to the Declaration of Helsinki and received the approval of the institutional Ethics Committee of the University of Minho (Braga, Portugal) and *Hospital de Braga.* The authors assert that all procedures contributing to this work comply with the ethical standards of the relevant national and institutional committees on human experimentation and with the Helsinki Declaration of 1975, as revised in 2008.

### Psychometric assessment

All participants completed the validated Portuguese versions of the obsessive-compulsive inventory (OCI), an 18-item inventory measuring six groups of symptoms (washing, checking, ordering, hoarding, obsessing, and neutralizing) [19, 20], and the emotion regulation questionnaire (ERQ), a tool assessing habitual use of two emotion regulation strategies: reappraisal and suppression [21, 22]. Additionally, OCD patients completed the Yale-Brown obsessive-compulsive scale (Y-BOCS) to measure symptom severity [23, 24].

### Imaging data acquisition

Data were acquired on a 3.0 Tesla clinical MRI scanner (Siemens Verio, Erlangen, Germany), equipped with a 32-channel head coil. All participants performed a cognitive reappraisal task inside the scanner (see below), during which we acquired a multi-band echo-planar imaging (EPI) sequence, CMRR EPI 2D (R2016A, Center for Magnetic Resonance Research, University of Minnesota, Minneapolis, MA) sensitive to fluctuations in the Blood Oxygenation Dependent Level (BOLD) contrast, with the following parameters: TR = 1000 ms, TE = 27 ms, FA = 62°, 2 mm^3^ isometric voxel size, 64 axial slices over a matrix of 200 *×* 200 mm^2^. This acquisition lasted for 7.8 min. The scanning session also included an anatomical gradient echo Magnetization-Prepared rapid acquisition in the sagittal plane (MPRAGE, repetition time [TR] = 2420 ms, echo time [TE] = 4.12 ms, flip angle [FA] = 9°, field of view [FOV] = 176 *×* 256 *×* 256 mm^3^, 1 mm^3^ isometric voxel size).

### fMRI cognitive reappraisal task

We used a well-validated cognitive reappraisal task [25, 26], consisting in the presentation of series of blocks showing neutral or negative picture stimuli that participants must: (a) observe (to passively observe neutral pictures); (b) maintain (to actively focus on the emotions elicited by negative emotional pictures, sustaining them over time); or (c) regulate (to reappraise the emotions induced by the negative emotional pictures by virtue of cognitive reappraisal techniques previously trained). Before scanning, participants were trained in distancing and reinterpretation strategies. For instance, in front of pictures depicting disturbing scenarios, they were told to reappraise their emotions by elaborating thoughts such as: (a) the scene is not real (e.g., the people on the screen are actors); (b) the situation will likely get better with time; (c) the situation is not as grave as it first appears (e.g., seeing the situation in a more positive light); and (d) the situation concerns unknown people and will not affect oneself. Participants were specifically instructed that they were not to use non-cognitive strategies (i.e., as looking away) during stimulus presentation. Picture stimuli were obtained from the International Affective Picture System [27] and were presented through an MRI-compatible angled mirror system (Lumina–Cedrus Corporation).

The task consisted of 12 blocks: four blocks for each condition. Conditions were pseudorandomized across the task to avoid the induction of sustained mood states. At the beginning of each block, a word (i.e., observe, maintain, or regulate) appeared in the middle of the screen for 4 s to provide instructions to participants for the upcoming block. After the prompt, participants viewed two different pictures of equal valence for 10 s each. After the presentation of the second picture, the intensity of the negative emotion experienced was self-rated by participants on a 1–5 number scale that appeared for 5 s (1 being “neutral” and 5 being “extremely negative”). Subjects provided these responses through an MRI-compatible response pad (Lumina–Cedrus Corporation). Each block was followed by 10 s of baseline during which a cross fixation was presented to minimize carryover effects.

### fMRI preprocessing and ICA

The functional images were preprocessed using fMRIPrep 1.4.1 [28] (RRID:SCR_016216), which is based on Nipype 1.2.0 [29, 30] (RRID:SCR_002502). A thorough description of the preprocessing pipeline can be found in the Supplementary Material. Regarding in-scanner movements, our exclusion criterion was a framewise displacement >0.5. Nevertheless, none of the participants surpassed this threshold, and therefore, no participants were excluded because of this reason. Additionally, a visual inspection of fMRIPrep output reports was performed to identify movement outliers and assess the accuracy of the coregistration.

Group ICA [31] was performed with the Gift toolbox (v3.0c) using the Infomax algorithm [32]. Before ICA, voxel intensity was normalized, and data from all participants were pooled into a single data set through a two-step data reduction approach using principal component analysis to enable the analysis of large data sets. Twenty-nine independent components were obtained after a free ICA analysis. Fifty ICA iterations were performed by ICASSO [33] to ensure stability of the estimated components. Finally, individual component maps and time courses were estimated using a group ICA 3 back-reconstruction approach. Because the ICA approach may identify noisy components corresponding to non-biological signal, such as movement artifacts, independent components of interest were selected after visual inspection of their spatial distribution [34]. Specifically, components that were mainly present in regions that do not generate BOLD signal (white matter, ventricles, or outside the brain) were excluded from the analysis. In addition, to further refine component selection, a correlation with the component templates distributed by the Functional Imaging in Neuropsychiatric Disorders Lab (https://findlab.stanford.edu/functional_ROIs.html) was performed, using the Gift toolbox.

### Statistical analyses

#### Behavioral data analyses

These analyses were conducted using SPSS v. 27 (IBM Corp; Armonk, NY). p-Values under 0.05 were considered statistically significant. Groups were compared on continuous variables using independent-sample *t*-tests or Mann–Whitney tests depending on the normality of the data. Sex/gender distribution between groups was analyzed using a chi-squared test. A 2 × 3 repeated-measures ANOVA was used to compare the intra-scanner ratings of each condition (observe, maintain, and regulate) between both groups. Moreover, participants’ self-reported success in lowering their intra-scanner negative emotion intensity was calculated by subtracting regulate ratings from maintain ratings (success = maintain − regulate), while participants’ reactivity during emotional processing was computed as reactivity = maintain − observe.

#### Statistical analysis of the component spatial maps

To determine brain areas significantly related at the whole-sample level to each component time course, second-level one-sample *t*-tests were performed with Statistical Parametric Mapping (SPM12). Significance threshold was set at *p* < 0.05, family-wise error corrected for multiple testing. As per our hypotheses, analyses were focused on networks of interest encompassing the frontal cortex, which were visually identified in the results from the one-sample *t*-tests: the frontoparietal networks (dorsal, right, and left), the default mode network, the salience network, and the frontotemporal network.

#### Statistical analysis of component time courses

To study how functional networks of interest were modulated by cognitive reappraisal, GLM was applied on each subject’s component time courses using a design matrix representing the task. This yielded a set of beta-weights representing the modulation of component time courses by the GLM regressors. The GLM design matrix used in these analyses included separate regressors to model each of the conditions (observe, maintain, and regulate), which were convolved with the hemodynamic response function. Time derivatives and parameters that modeled residual motion were also included. Then, we performed separate second-level group analyses for the contrasts maintain > observe and regulate > maintain using the estimated beta-weights.

These group comparisons were performed in SPSS by means of a GLM including group (OCD patient or control) as a fixed factor. Normality (Kolmogorov–Smirnov, or K-S) tests were performed to assure that components were normally distributed. Age was modeled as a nuisance covariate due to its known modulatory effects on emotion regulation networks [35, 36]. A false discovery rate (FDR) approach was used to correct for the number of networks.

#### Brain–behavior correlations

Linear associations between network activations and all behavioral scales scores (6 OCI, 2 ERQ, and 3 Y-BOCS subscales) were assessed using Pearson correlations in SPSS. We also explored the associations between imaging and intra-scanner success ratings. These correlations were performed both for the full sample and for each group separately at an exploratory, uncorrected, threshold.

## Results

### Sociodemographic and clinical characterization

Both groups were comparable in terms of age, years of education, and sex/gender (Table 1). The clinical information for the OCD group (age of onset, symptom severity, and medication status) is also shown in Table 1.

### Behavioral results

#### Outside-scanner behavioral measures

There were no significant between-group differences on ERQ scores. Conversely, patients with OCD scored significantly higher in global and all symptom-specific OCI scores, except for the Hoarding score (Table 1).

#### Intra-scanner ratings

We used a 2 × 3 repeated-measures ANOVA to compare the intra-scanner ratings of each condition (observe, maintain, and regulate) between groups; since the assumption of sphericity was violated, the Huynh–Feldt correction was used. We observed a significant main effect of condition (*F*(1.711, 97.542) = 102.239, *p* < 0.001), with post-hoc tests showing that maintain ratings differed from observe ratings, which indicated successful negative emotion induction during this condition for the whole sample (*t* = −13.815, *p*_holm_ < 0.001). Regulate scores also differed from maintain scores, indicating successful emotion regulation (*t* = 3.709, *p*_holm_ < 0.001). There was no main effect of group (*F*(1, 57) = 0.043, *p* = 0.836), nor any interactions between group and condition (*F*(1.711, 97.542) = 2.299, *p* = 0.114). Nevertheless, the success variable significantly differed between the study groups (*t*(57) = 2.34, *p* = 0.023), with HC showing more successful regulation, while there were no significant between-group differences in the reactivity variable.

### ICA results

**Figure 1.**
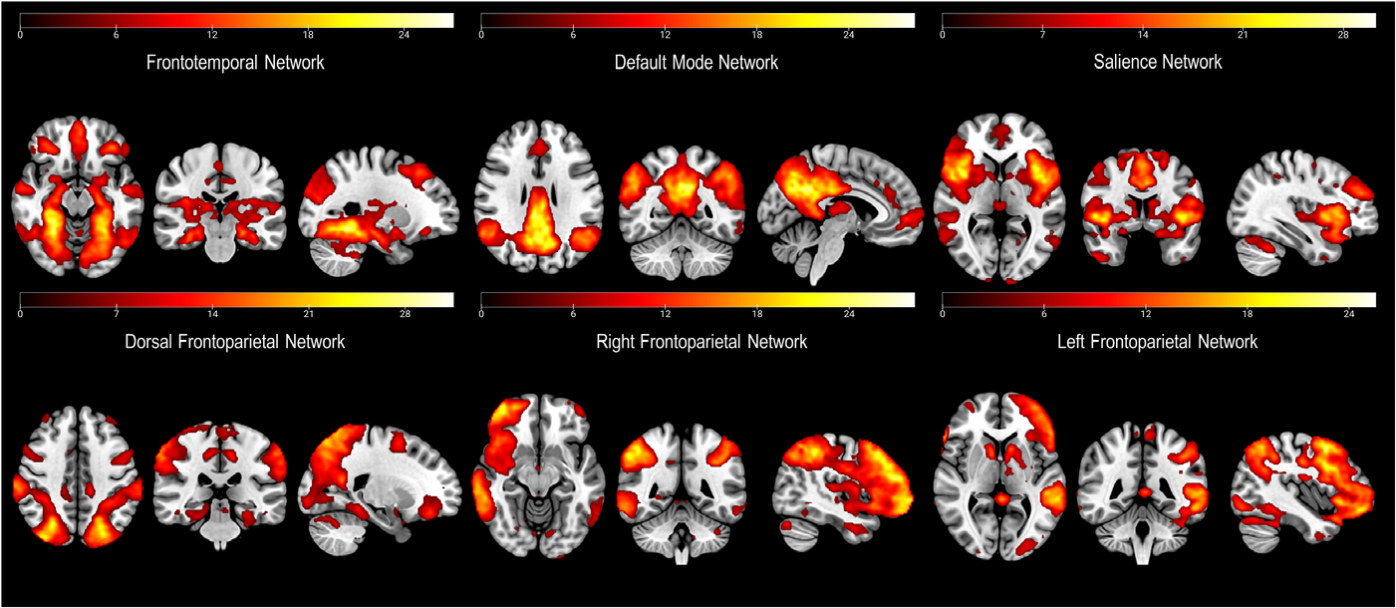
Depiction of the networks of interest (i.e., including parts of the frontal cortex) derived from the independent component analysis (ICA).

Out of the 29 components obtained, we excluded 18 of them due to a lack of correlation with any recognizable network. The remaining 11 networks were identified as primary visual, language, secondary visual, cerebellum, salience, auditory, default mode, left frontoparietal, dorsal frontoparietal or dorsal attention network (DAN), right frontoparietal, and frontotemporal network. As per our hypotheses, from these networks we selected those encompassing frontal cortex regions (Figure 1).

Brain regions characterizing each network can be seen in Figure 1. Most of these networks are those usually identified during the resting state (37). Nevertheless, we also identified a less common frontotemporal network, which included medial temporal lobe structures (including the amygdala), and cortical areas such as the bilateral fusiform gyri (FG), the middle and inferior frontal gyri (MFG, IFG), the angular gyrus (AG), the claustrum, the middle and superior temporal gyri (MTG, STG), the precuneus, the anterior cingulate cortex (ACC), and the precentral gyrus (PG) (Figure 1).

### Analysis of components time courses

**Figure 2.**
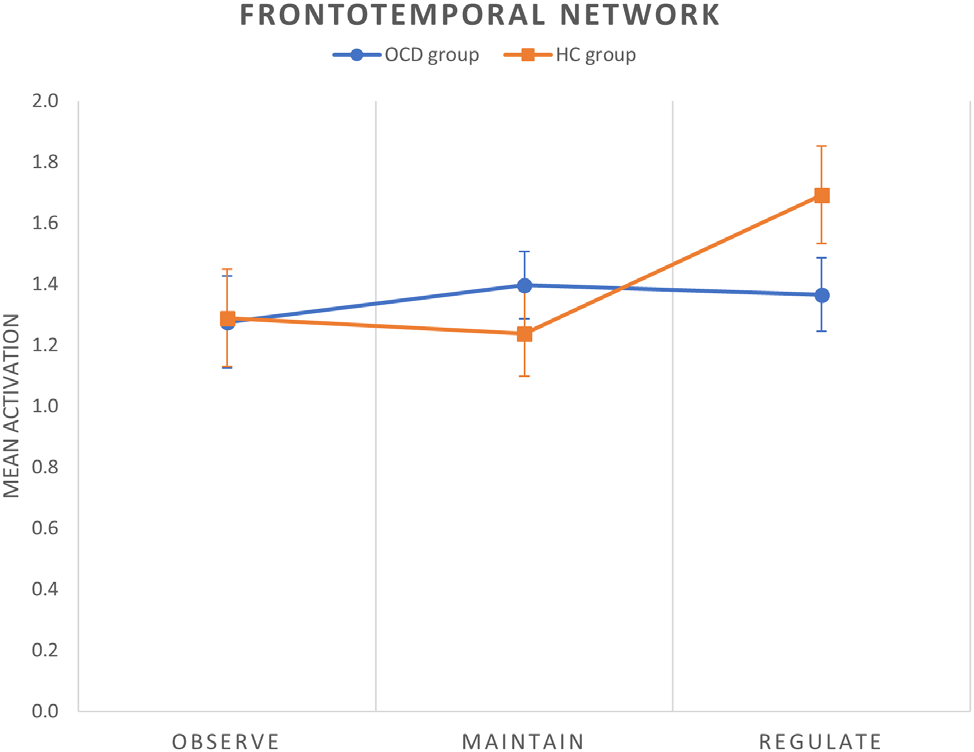
Mean group activations during each condition of the cognitive reappraisal task in the frontotemporal network. Error bars indicate standard error of the mean.

**Figure 3.**
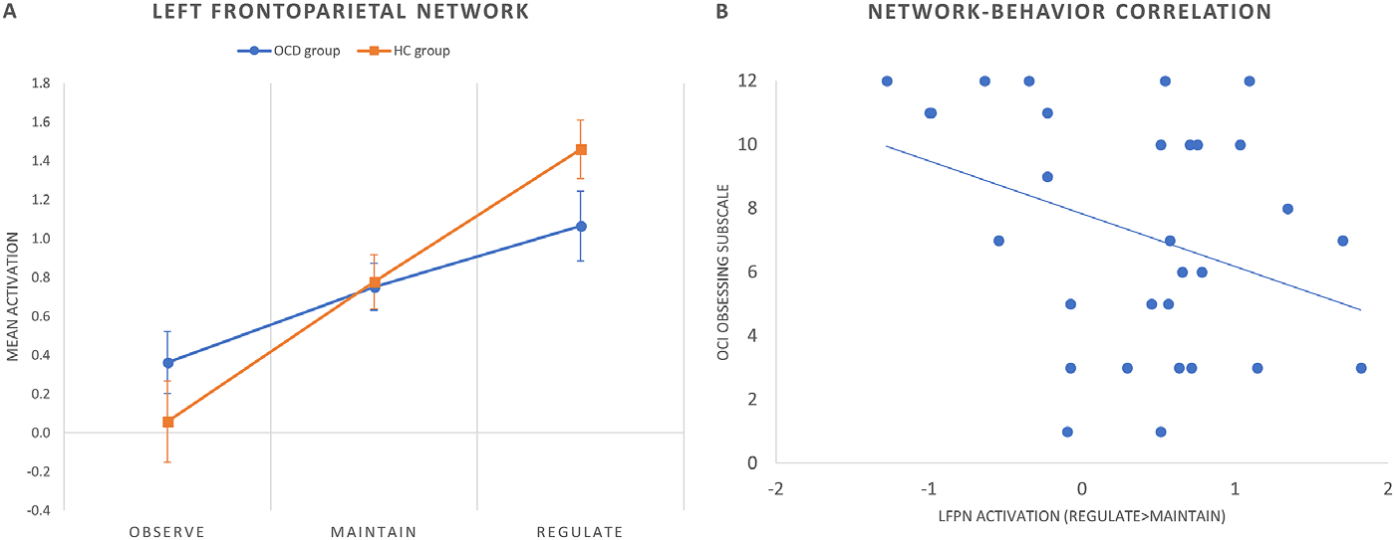
(A) Mean group activations during each condition of the cognitive reappraisal task in the left frontoparietal network (LFPN). Error bars indicate standard error of the mean. (B) Scatter plot (and linear trend) depicting the correlation between OCI obsessing subscale scores and LFPN activation (regulate > maintain contrast) in patients with OCD.

We obtained two different results in the regulate > maintain contrast. All the components were normally distributed (all *p* values of the K-S test >0.05). We observed a between-group difference within the frontotemporal network, with patients with OCD showing a decreased activation of this network during cognitive reappraisal (corrected model: *F*(2,58) = 5.84, *p* = 0.005, *p*_FDR-corr_ = 0.030; group effect: *F*(1,58) = 7.81, *p* = 0.007) (Figure 2). Moreover, within the left frontoparietal network (LFPN) we observed a trend-level decreased activation in patients with OCD during reappraisal (corrected model: *F*(2,58) = 3.99, *p* = 0.024, *p*_FDR-corr_ = 0.072; group effect: *F*(1,58) = 3.60, *p* = 0.063). These results are displayed in Figure 3A.

No significant results were observed in the maintain > observe contrast.

### Brain–behavior correlations

We assessed Pearson’s correlations between clinical variables and brain activity within the frontotemporal and LFPNs. Within the OCD group, LFPN (regulate > maintain) activity correlated negatively with the OCI obsessing subscale score (Pearson’s *r* = −0.407, *p* = 0.029) (Figure 3B). No further correlations were observed with psychometric scores or intra-scanner success ratings.

## Discussion

Alterations in emotion regulation capacities contribute, to a varying extent, to the symptom profile of most neuropsychiatric disorders [3, 38]. Recent research using dynamic casual modeling has found that dynamic interactions between frontal regions and the amygdala form a recursive feedback loop, which determines the effectiveness of emotion-regulatory actions [39]. OCD is no exception to this rule, and it has been indeed considered to be a disorder of self-regulation and behavioral inhibition, which may be accounted for by alterations in the recurrent projections linking the frontal cortex with subcortical structures [40]. Beyond cortico-striatal circuits, such fronto-subcortical projections also involve fronto-amygdalar connections, implicated in the modulation of fear and anxiety symptoms in patients with OCD [41]. In this study, we focused our analyses in networks encompassing the frontal cortex, since it has been shown that neurobiological underpinnings of emotion regulation alterations typically involve blunted responses in frontal areas during cognitive reappraisal [4]. Here, we show that the network displaying larger alterations in OCD was the one linking prefrontal regions, such as the ventrolateral and dorsolateral prefrontal cortices -which typically show decreased activations in clinical populations during cognitive reappraisal in activation-based studies [3]-, with medial temporal lobe structures (i.e., the amygdala) and other temporal and parietal cortical areas. Our results therefore align with the previous literature highlighting the importance of prefrontal-limbic disruptions for OCD.

The decreased values observed in the frontotemporal network in the OCD sample should be carefully interpreted. These values refer to the average signal across the different regions of the network, which involve cortical (frontal, temporal and parietal) and subcortical structures (i.e., amygdala). At first sight, our findings may seem in apparent contradiction with studies describing heightened limbic activation and increased connectivity between fusiform and dlPFCs and the amygdala during emotional processing in OCD [11]. Those results were nevertheless obtained during an emotional face processing paradigm, and not during voluntary emotion regulation. Indeed, it has been reported that the regions of the frontotemporal network may display differential patterns of activation across different phases of emotional processing in patients with OCD [10]. Specifically, this study showed increased amygdala reactivity to negative stimuli and decreased dlPFC engagement together with a diminished frontal-amygdala connectivity during emotion regulation. Although our network approach does not allow for assessing such differential activations across the different regions of the frontotemporal network, the overall decreased network activity reported here may be interpreted as related to a decreased engagement during cognitive reappraisal of the wide area of cortical regulatory regions. This, in turn, could trigger dysregulated activity in the amygdala. Furthermore, our results in the frontotemporal network can be related to the structural white matter alterations, in terms of decreased fractional anisotropy in clusters within the uncinate fasciculus, observed in OCD patients with a diffusion tensor imaging approach [42].

Obsessive-compulsive symptoms have been traditionally linked to alterations in cortico-striatal circuits [43]. It may therefore be questioned to what extent the frontolimbic alterations may contribute to core disorder’s symptoms or merely have an impact on unspecific fear and anxiety symptoms. In this sense, it has been shown that disrupted emotion regulation may lead to obsessive-compulsive symptoms through indirect pathways involving alterations in positive affect and decreased cognitive flexibility [44]. Likewise, the inability to downregulate emotions may lead to the deployment of suppression strategies, which have been linked to the occurrence of obsessive thoughts [45]. Moreover, at the neural level, dysregulated amygdala input to the prefrontal cortex has been shown to disrupt cognitive processes depending on cortical-striatal circuitry in patients with OCD [46].

The trend-level decrease in left FPN activity also described in our OCD sample can be interpreted in similar terms. This network comprises cortical frontoparietal regions, which have been shown to participate in the downregulation of emotions [4, 47] and partially overlap with those included in the frontotemporal network, as well as some subcortical clusters mainly located in ventral striatal areas. Interestingly, this left FPN activity decrease correlated with obsessing symptoms. In previous studies from our group, we have shown that these symptoms correlate with increased amygdala reactivity to negative stimuli [48], as well as with a reduced connectivity between the ventral striatum and limbic regions [49]. Present results seem to support these previous findings suggesting that increased amygdala reactivity observed in individuals with obsessing symptoms stems from an inefficient control from cortical frontoparietal and striatal regions.

This study is not without limitations. First, although the network approach allows characterizing brain activity during cognitive reappraisal in terms of patterns of regions of coordinated activity, and, therefore, in terms of functional brain units, it lacks specificity regarding putative activation differences across the regions of the network. Second, emotion regulation success was exclusively assessed with subjective intra-scanner ratings, which have shown lower reliability and validity in previous studies [3]. Future studies should consider including other approaches, such as psychophysiological measurements, to overcome this issue. Finally, most of the patients were medicated, creating a potential effect that cannot be isolated and could bias results.

In sum, this study indicates that patients with OCD show decreased activation of frontotemporal and frontoparietal networks during cognitive reappraisal, which can eventually lead to limbic hyperreactivity in front of aversive stimuli. Such emotion regulation difficulties can not only increase unspecific fear and anxiety symptoms but also interact with the expression of core OCD symptoms. Our results should help to better characterize the psychological processes modulating the clinical profile of patients with OCD, as well as the associated neurobiological alterations. Moreover, the network approach used in this study allows the description of brain alterations from a system-level perspective, which is aligned with recent accounts on the effects and mechanisms of action of different treatment strategies for OCD [50–52]. Further research on the predictive value of network-level activity on treatment response—including pharmacological, psychological, and neuromodulation treatments—is therefore warranted. Likewise, it will also be important to develop treatment approaches aimed at modulating network activity. In this sense, the system-level effects of regulating neural activity within discrete regions, such as in deep brain stimulation and other neuromodulation approaches, should be assessed, while incipient neuromodulation techniques, such as fMRI-based neurofeedback, may be probably developed with the aim of regulating network-level activity.

## Data Availability

The data that support the findings of this study and the brain maps of all analyses are available from the corresponding author upon reasonable request.

## Supplementary material

For supplementary material accompanying this paper visit https://doi.org/10.1192/j.eurpsy.2022.2322. click here to view supplementary material
